# Clustering and Correlations amongst NEFA, Selected Adipokines and Morphological Traits—New Insights into Equine Metabolic Syndrome

**DOI:** 10.3390/ani12202863

**Published:** 2022-10-20

**Authors:** Zsofia Daradics, Mihaela Niculae, Cristian Mihăiță Crecan, Alexandru Florin Lupșan, Mirela Alexandra Rus, Sanda Andrei, Dana Mihaela Ciobanu, Florinela Adriana Cătoi, Ioana Delia Pop, Mircea Valerian Mircean, Cornel Cătoi

**Affiliations:** 1 Department of Pathology, Faculty of Veterinary Medicine, University of Agricultural Sciences and Veterinary Medicine, 400372 Cluj-Napoca, Romania; 2 Department of Infectious diseases, Faculty of Veterinary Medicine, University of Agricultural Sciences and Veterinary Medicine, 400372 Cluj-Napoca, Romania; 3 Department of Anaesthesiology and Surgery, Faculty of Veterinary Medicine, University of Agricultural Sciences and Veterinary Medicine, 400372 Cluj-Napoca, Romania; 4 Department of Reproduction, Obstetrics and Veterinary Gynaecology, Faculty of Veterinary Medicine, University of Agricultural Sciences and Veterinary Medicine, 400372 Cluj-Napoca, Romania; 5 Department of Biochemistry, Faculty of Veterinary Medicine, University of Agricultural Sciences and Veterinary Medicine, 400372 Cluj-Napoca, Romania; 6 Department of Diabetes and Nutrition Diseases, Faculty of Medicine, Iuliu Hatieganu University of Medicine and Pharmacy, 400012 Cluj-Napoca, Romania; 7 Department of Pathophysiology, Faculty of Medicine, Iuliu Hatieganu University of Medicine and Pharmacy, 400012 Cluj-Napoca, Romania; 8 Department of Exact Sciences, Faculty of Forestry and Cadastre, University of Agricultural Sciences and Veterinary Medicine, 400372 Cluj-Napoca, Romania; 9 Department of Internal Medicine, Faculty of Veterinary Medicine, University of Agricultural Sciences and Veterinary Medicine, 400372 Cluj-Napoca, Romania

**Keywords:** adipokines, non-esterified fatty acids, cresty neck score, body condition score, horse, metabolic syndrome

## Abstract

**Simple Summary:**

While adipokines’ involvement as biomarkers is better established in human pathology, little data are available on horses. This study aimed to investigate the possible association and relationship between selected metabolic parameters and morphological traits in equine metabolic syndrome. Spearman correlation, univariate linear regression, and hierarchical clustering were used to analyze the correlation between total cholesterol, insulin, non-esterified fatty acids (NEFA), adipokines and age, bodyweight, and general and local adiposity scores. NEFA correlated positively with low significance with bodyweight and adiposity scores, total cholesterol with bodyweight, and omentin with adiposity score. Cluster analysis supported these results and provided further insight into the relationships between studied variables within and between the four groups. These findings highlight NEFA, chemerin, and omentin as valuable biomarkers that could be further analyzed in other horse breeds for a better understanding of equine metabolic pathology.

**Abstract:**

Obesity is a common feature in horses suffering from metabolic syndrome. While adipokines involvement as biomarkers is better established in human pathology, little data are available on horses. This study aimed to investigate the possible association and relationship between selected metabolic parameters and morphological traits in equine metabolic syndrome. Adiposity was evaluated using body condition score (BCS) and cresty neck score (CNS). Plasma levels of total cholesterol, insulin, NEFA, and adipokines (omentin and chemerin) were determined using enzyme-linked immunosorbent assays. Spearman correlation, univariate linear regression analysis and hierarchical clustering were performed. Significant positive correlations were observed between NEFA and bodyweight (r = 0.322; *p* = 0.006), BCS (r = 0.295; *p* = 0.013), and CNS (r = 0.267; *p* = 0.024), total cholesterol and bodyweight (r = 0.262; *p* = 0.027), and omentin and CNS (r = 0.234; *p* = 0.049). Cluster analysis supported these results and provided more details on the relationships between studied variables within and between the four resulting groups. These findings highlight NEFA, chemerin, and omentin as valuable biomarkers that could be further analyzed in other horse breeds for a better understanding of equine metabolic pathology.

## 1. Introduction

Obesity is a major health problem in both animals and humans, and it is linked to metabolic syndrome, typically characterized by glucose intolerance, dyslipidemia, and hypertension [1,2,3]. Triggered by macronutrients accumulation, the adipose tissue produces and releases both pro-inflammatory mediators, such as tumor necrosis factor α alpha (TNF), interleukin (IL)-6, IL-18, resistin, leptin, and retinol-binding protein 4 (RBP4), as well as anti-inflammatory mediators, such as adiponectin and secreted frizzled-related protein 5 (SFRP5) [4]. Excess adipose tissue has been associated with the increased production of TNF and IL-6, and the downregulated production of other substances, such as adiponectin and SFRP5 [5,6,7]. The imbalance of these adipokine levels may result in the development of obesity-related metabolic disorders. However, these mechanisms and aspects are poorly investigated in animals. Circulating IL-6, IL-1β, and TNF appear to increase with obesity in mice, cats, and dogs [8,9,10]. In horses, initially, only the correlation between TNF and body condition score (BCS) was reported [11], while the links with the interleukins were pointed out by a later study [12].

Omentin is mainly expressed in stromal vascular cells and is associated with anti-inflammatory, anti-atherogenic, anti-cardiovascular, and antidiabetic properties [13,14,15]. Omentin has been positively associated with adiponectin and high-density lipoprotein levels, and negatively related to bodyweight, waist circumference, and insulin resistance [16,17]. Low plasma omentin levels contribute to insulin resistance, type 2 diabetes, and cardiovascular diseases in obese or overweight patients [18,19]. Chemerin secreted by the adipose tissue is one of the recently identified adipokines and plays an important role in adipogenesis, glucose metabolism, and inflammation [20,21]. While adipokine’s involvement as biomarkers is better established in human pathology, little data are available on horses.

Non-esterified fatty acids (NEFA) are released from triglycerides (TG) and are an important source of energy for many tissues [22,23]. Obese individuals are exposed to an increased level of circulating NEFAs. These elevated levels have been associated with insulin resistance and physiological disturbances [24,25,26]. A negative energy balance induces excessive fat mobilization, resulting in decreased liver function and the accumulation of NEFA [27]. The increased release of NEFA from visceral adipose tissue affects hepatic insulin elimination and causes systemic hyperinsulinemia [28].

In equids, the dorsal neck region is one of the most susceptible anatomic parts for fat deposition, and neck obesity in horses is more prevalent than overall obesity [29]. Moreover, excess fat deposition in the neck region has been linked with insulin resistance and an elevated risk of laminitis [30,31].

To our knowledge, this is the first study aimed to evaluate the relationship between selected metabolic variables (total cholesterol, NEFA, and adipokines) and general and local adiposity scores in horses.

The aim of this study was to investigate the possible association between selected metabolic parameters and morphological traits in horses with metabolic syndrome.

## 2. Materials and Methods

### 2.1. Study Design and Animals

This cross-sectional study was performed on local Romanian draft horses from Northwestern Romania. Seventy-one animals matching criteria according to the latest consensus statement issued by the American College of Veterinary Internal Medicine (ACVIM) [32], namely (1) generalized obesity or increased regional adiposity, (2) evidence of insulin resistance, hyperinsulinaemia or exaggerated insulin responses to oral glucose, and (3) a predisposition toward laminitis, were included.

The use of animals in this study involved the evaluation of selected physiological and metabolic parameters. These procedures were reviewed and approved by the Bioethics Committee of the University of Agricultural Sciences and Veterinary Medicine Cluj-Napoca, Romania (approval number 224/20 August 2020). The horse owners contributed with their written consent.

For each animal included in the study, clinical examination was performed by a single experienced veterinarian to assess two parameters: (1) BCS and (2) cresty neck score (CNS) using the nine-point Henneke scale [33] and the five-point Carter scale [34], respectively. With the Henneke scale, a horse or pony can be classified as overweight with a BCS of 7 and obese when the BCS is ≥8 [33]. The Carter scale evaluates the local deposition of fat tissue (nuchal crest) and it ranges from 0 (no crest) to 5 (large crest dropping to one side), with a score of ≥3 indicating a cresty neck [34].

Additionally, blood samples (6 mL) were collected via jugular vein puncture using sterile K3 EDTA tubes (Greiner Bio-One GmbH, Kremsmünster, Austria), then centrifuged at 1800× *g* at 4 °C for 15 min. The plasma samples were stored in 1.5 mL Eppendorf tubes at −20 °C until analysis.

### 2.2. Analytical Methods

The quantitative determination of NEFA, total cholesterol, omentin and chemerin in horse plasma was performed by enzyme-linked immunosorbent assays (ELISA) at the Laboratory of Immunochemistry of the Faculty of Veterinary Medicine Cluj-Napoca, Romania. As in previous reports [35], commercially available ELISA kits specific for equine species (https://www.mybiosource.com/equine-elisa-kits accessed on 1 March 2022) were used following the manufacturers’ instructions as follows: Horse Non-esterified fatty acid (NEFA) Elisa Kit (MyBioSource, Inc., San Diego, CA, USA, Catalog No: MBS778992), Horse Total Cholesterol ELISA kit (MyBioSource, Inc., San Diego, CA, USA, Catalog No: MBS1602038), Horse Insulin (INS) ELISA kit (MyBioSource, Inc., San Diego, CA, USA, Catalog No: MBS280388), Horse Omentin (OMT) ELISA kit (MyBioSource, Inc., San Diego, CA, USA, Catalog No: MBS024519), and Horse Chemerin (CHE) ELISA kit (MyBioSource, Inc., San Diego, CA, USA, Catalog No: MBS022438). All ELISA kits used are commercially available from MyBioSource to be used for horse samples only. As recommended by manufacturers’ instructions, all standards and plasma samples were run in duplicate. Optical density was measured using a microplate reader Sunrise™ (Tecan, Männedorf, Switzerland), and the results were analyzed using the Magellan™ software. The plasma levels of these parameters were calculated based on the standard curves generated by plotting the optical density of standards against the concentrations.

According to the manufacturer, these ELISA kits were validated, and the quality control assessing the reproducibility identified the intra-assay CV (%) and inter-assay CV (%) with these characteristics, presented in Table 1. The intra- and inter-assay coefficients of variability (CV) were calculated and found to meet the manufacturer criteria: NEFA 9.3% and 13.2%, respectively; total cholesterol 7.3% and 9.4%, respectively; insulin 7.7% and 8.6%, respectively; omentin 5.8% and 8.6%, respectively; and chemerin 5.04% and 5.6%, respectively.

### 2.3. Statistical Analysis

Data analysis was performed using the IBM SPSS Statistics and Microsoft Office for Windows, Version 22.0 (Armonk, NY, USA: IBM Corp). The Shapiro–Wilk test was used to assess all variables’ normal distribution. Data were expressed as mean ± standard deviation, numbers and percentages, or median (25th, and 75th percentiles). Correlations between variables were evaluated using a non-parametric Spearman rank coefficient, with the value of *p* < 0.05 being considered statistically significant. Univariate linear regression analysis adjusted for age and sex was performed to assess the association of NEFA (as a dependent variable) with bodyweight and cresty neck score. In addition, hierarchical clustering was conducted using NEFA, omentin, and chemerin values to globally evaluate the variation of these parameters as follows: values were transformed in ranks *z* using statistical indicators m and σ and a three-component vector was associated with each horse. Based on standardized Euclidean distances, horses were included in four groups in a way that values of the three variables are as similar within group and as different between groups as possible. The dendrogram of hierarchical clustering was performed using the complete linkage method. Values for age, bodyweight, NEFA, omentin, chemerin, total cholesterol, and insulin were further compared using one-way ANOVA analysis. When significant differences were observed between groups, Hochberg GT2 and Games–Howell post-hoc tests were performed.

## 3. Results

### 3.1. Characteristics of Study Population

In total, 71 horses (local Romanian draft horse breed) were evaluated. Of these, 38.0% were males, with a median age of 9.0 years and a median weight of 600.0 kg. The mean plasma level of NEFA was 420.1 μmol/L, while the median values for total cholesterol and insulin were 2.9 mmol/L, and 55.4 μIU/mL, respectively. The median plasma adipokines omentin and chemerin levels were 14.3 ng/mL and 283.0 ng/mL, respectively (Table 2). General adiposity evaluated by the Henneke scale pointed out BCS of 7 in 22 (31.0%) of 8 in 26 (36.6%) and of 9 in 23 (32.4%). For 29 horses (40.8%), CNS was 5, while 23 (32.4%) had a score of 9 4 and 19 (26.8%) had a score of 3.

### 3.2. Correlations

**Figure 1:**
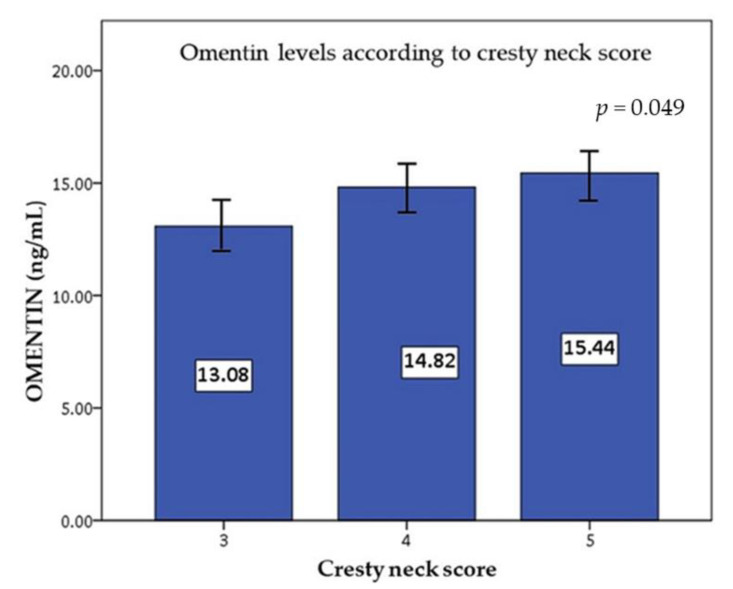
Omentin levels according to cresty neck score.

NEFA was significantly (*p* < 0.05) associated with bodyweight, age, and with BCS and CNS (Table 3). Total cholesterol presented a significant positive correlation (r = 0.262; *p* < 0.05) with bodyweight. Insulin was not associated with any of these parameters (*p* > 0.05). The correlations displayed weak positive, but significant relationships. Omentin was significantly associated with CNS (r = 0.234; *p* < 0.05) (Table 2, Figure 1), while chemerin did not present with any significant associations (*p* > 0.05).

### 3.3. Linear Regression Analysis

**Figure 2:**
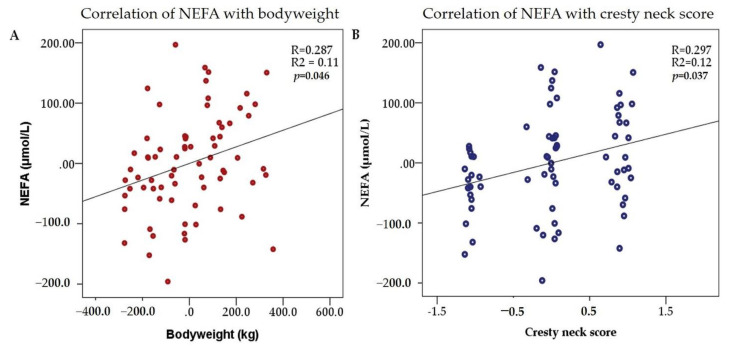
Correlation between NEFA and bodyweight (**A**) and cresty neck score (**B**). The dots represent correlations between the 2 parameters.

When NEFA was included in the univariate linear regression analysis as the dependent variable, we found that the bodyweight (Model 1) and CNS (Model 2) were independent predictors (Table 4, Figure 2). For a 1 kg increase in bodyweight, the NEFA level increases with 0.139 units. Similarly, a one-point increase in the CNS results in a NEFA level increase of approximately 32 units. The results were adjusted for age and sex. The resulting overall model fit were R^2^ = 0.11; *p* = 0.046 (Model 1) and R^2^ = 0.12; *p* = 0.037 (Model 2).

### 3.4. Cluster Analysis

**Figure 3:**
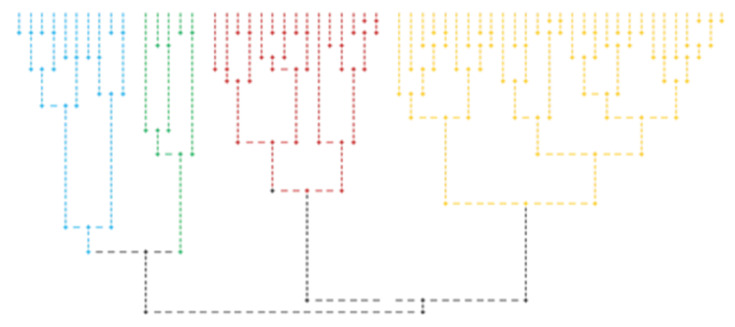
The dendrogram of hierarchical clustering using the complete linkage method and the resulting four groups (Group 1—yellow, Group 2—red, Group 3—blue, Group 4—green).

Hierarchical clustering analysis was performed to assess the variation of studied variables as follows: values for total cholesterol, insulin, NEFA, and adipokines (omentin and chemerin) were transformed into standardized ranks z using statistical indicators m and σ. A total of 59 horses were retained to be grouped, those who had all five z coordinates located in the range [−2, +2]. We aimed to group the horses so that within each group, the horses were as similar as possible regarding the values of the variables NEFA, omentin, and chemerin, and the horses from any two groups were as different as possible from each other in terms of the values of the three variables. Thus, a three-component vector was associated with each horse. Based on standardized Euclidean distances, four groups were formed: Group 1 (29 horses), Group 2 (15 horses), Group 3 (10 horses) and Group 4 (5 horses) (Figure 3).

The comparison of variables by one-way ANOVA showed that bodyweight, NEFA, omentin, chemerin, and total cholesterol values differ significantly between groups (*p* < 0.05) (Table 5). When the differences between the means were found statistically significant at a *p* < 0.05, the means were further compared between two groups. These comparisons were made using Hochberg’s GT2 test for weight, omentin and total cholesterol and by Games–Howell test for NEFA and chemerin, respectively. Statistically significant differences were noticed between Group 1 and Group 4 (*p* = 0.001), Group 1 and Group 2 (*p* = 0.015) for bodyweight, between Group 1 and Group 2 (*p* < 0.001), Group 1 and Group 4 (*p* = 0.008), Group 2 and Group 3 (*p* = 0.029) for NEFA, between Group 1 and Group 4 (*p* < 0.001), Group 2 and Group 4 (*p* = 0.001), Group 3 and Group 4 (*p* = 0.032) for omentin, Group 1 and Group 3 (*p* < 0.001), Group 1 and Group 4 (*p* = 0.009), Group 2 and Group 3 (*p* < 0.001), Group 2 and Group 4 (*p* = 0.006) for chemerin, and between Group 1 and Group 3 (*p* = 0.020) for total cholesterol (Table 5).

**Figure 4:**
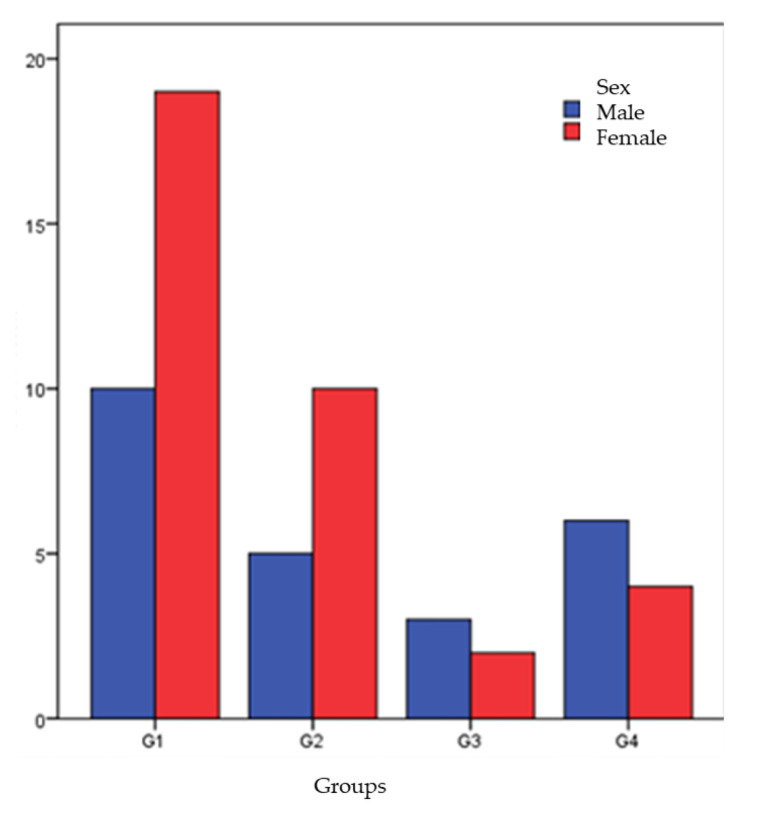
Sex distribution within the groups.

Horses were mostly mares in Group 1 and Group 2 and stallions in Group 3 and Group 4 (Figure 4).

**Figure 5:**
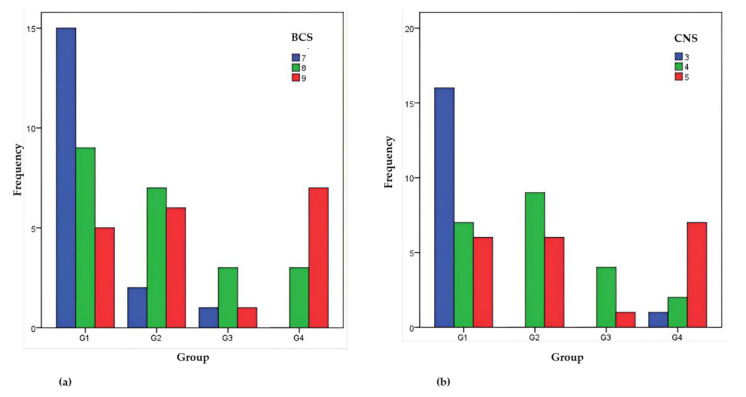
The frequencies of the horses in the four groups according to the BCS (**a**) CNS (**b**).

Most horses had BCS 7 and CNS 3 in Group 1, BCS 8 and 9 and CNS 4 in Group 2, BCS 8 and CNS 4 in Group 3, and BCS 9 and CNS 5 in Group 4 (Figure 5).

**Figure 6:**
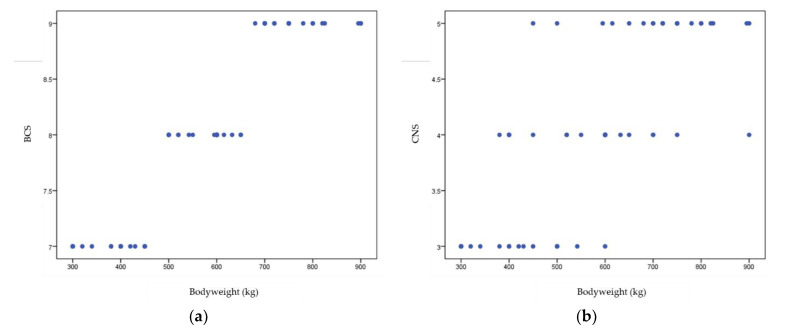
Correlation diagram of variables: bodyweight and BCS (**a**) and CNS (**b**).

The frequency distributions of BCS and CNS variables compared between the groups using the Kruskal–Wallis test differed significantly (*p* < 0.05) (Table 6). The lowest BCS values are in group G1, followed by those in group G3, and the highest in group G4. Likewise, the lowest CNS values are in group G1, followed by those in group G3, and the highest in group G4. The results indicated that low BCS and CNS values are more frequent in Group 1, while high BCS and CNS values are predominant in Group 4 correlated well (*p* < 0.001) with bodyweight that is lowest in Group 1 and highest in Group 4 (Table 7, Figure 6).

Furthermore, following the Kruskal–Wallis test, we also performed the post hoc Mann–Whitney U test which revealed significant differences: between Group 1 and Group 2 (z = 2.456; *p* = 0.014), between groups Group 1 and Group 4 (z = 3.384; *p* = 0.001) for BCS, between Group 1 and Group 2 (z = 3.002; *p* = 0.003) and between groups Group 1 and Group 4 (z = 2.899; *p* = 0.004) for CNS.

## 4. Discussion

The association between obesity, insulin resistance and metabolic syndrome has been extensively studied and is well described in the literature [25,28,36]. However, there are numerous underlying mechanisms and processes that are still controversial and have not been elucidated in both human and equine metabolic syndrome.

In the present study, possible associations and relationships between selected metabolic variables (total cholesterol, NEFA and adipokines) and general and local adiposity scores were assessed in horses suffering from metabolic syndrome.

Though most of the previous studies evaluating metabolic dysfunctions have been focused on obese animals, lean horses can also develop metabolic dysregulation and insulin dysfunction [37]. Available data are very limited; some possible explanations, however, are proposed, mostly relying on the altered hepatic response of affected lean horses to certain nutrients and increased production of cortisol within adipose tissue [36]. Therefore, this study was designed with a single group of horses. Furthermore, the local Romanian draft horse breed appears to be predisposed to develop equine metabolic syndrome, as their owners prefer higher BCS as health and welfare criteria (unpublished data).

The results of our study indicated positive correlation between NEFA levels and bodyweight, BCS, and CNS, while age was negatively correlated with NEFA levels. Linear regression analysis pointed out that bodyweight and CNS are independent predictors of NEFA plasmatic levels. Our findings suggest that there are weak positive, but significant relationships between the described parameters.

In obese insulin resistant horses, metabolic disorders are accompanied by decreased liver capacity to convert NEFAs to TGs and incorporate TGs in very-low density lipoprotein (VLDL), leading to a significant increase in serum NEFA concentration. Previous studies have confirmed that NEFA, VLDL, and high-density lipoprotein-cholesterol levels are significantly higher in obese insulin resistant horses than in lean horses without insulin resistance [12,31], which might be related to increased lipoprotein lipase activity [38]. In healthy ponies and horses subjected to progressive weight gain, BCS and CNS increased significantly in the first and second year of excessive energy intake as compared to the baseline. Serum NEFA and insulin concentrations were higher in the second year than at baseline in ponies and did not change in horses [39]. On the contrary, other studies reported no effects of BCS or percent weight loss changes on serum TG and NEFA concentrations [40,41]. Although in our study we could not correlate insulin with other variables, there is a general agreement that serum insulin together with NEFA concentrations, might be good parameters for following up animals prone to obesity and insulin dysfunction.

Since insulin was not significantly associated with any of the analyzed parameters in this study, our analysis might indicate that metabolic syndrome is likely to be caused by the alteration of lipid metabolism rather than the glucose pathways. Available literature regarding the association between insulin concentration and age or sex are controversial. In some studies, differences between sexes were found in plasma insulin concentrations [12,42]. Our results are in line with some recent studies where age- and sex-related differences in insulin concentrations were not observed [43,44]. In a study on 27 Andalusian horses that are known to be predisposed to fat accumulation in the neck, no differences associated with sex or age were found either in plasma insulin or leptin concentrations at the baseline. However, insulin concentrations were correlated with crest height and neck circumferences [43]. Another study showed that overconditioned and obese horses had higher plasma insulin and TG levels than optimally conditioned horses [44]. The impact of bodyweight changes on insulin sensitivity in obese equines is also the subject of debate. While intensive bodyweight loss (1% of bodyweight per week) has been reported to improve insulin sensitivity in ponies [36], other studies reported no such effect in either insulin-sensitive or insulin-resistant ponies under a bodyweight reduction program [45,46].

Total cholesterol correlated only with the bodyweight, which may suggest that clinical manifestations of metabolic disorders are primarily driven by other mechanisms, rather than hypercholesterolemia. However, our observation is in line with the study conducted by Ribeiro et al., in which Mangalarga Marchador horses fed on a hypercaloric diet experienced a significant increase in cholesterol after 30 days, and this increase was maintained for 150 days. Interestingly, NEFA values did not differ from the baseline [47].

In our study, a statistically significant association was observed between omentin level and CNS. The relationship between these adipokines and metabolic disorders has been investigated in humans, but data in animals are scarce. In horses, BCS and insulin levels have been rather correlated with proinflammatory cytokines such as TNF, IL-1β, and IL-6 [12]. Both TNF and IL-6 were positively correlated with sex, with concentrations being higher in mares, while TNF was negatively correlated with TG, and IL-6 was positively correlated with age [37]. In a study in cats, omentin was not affected by bodyweight or adiposity but plasma omentin concentrations were significantly greater (56%) in male than female cats, and no differences were observed between obese and ideal weight cats [48]. Moreover, obese cats had increased insulin, glucose, insulin:glucose ratio, and TG levels but similar cholesterol levels as normal weight cats. This study, in contrast with human studies, has not found a significant correlation between omentin levels and BCS, insulin, or glucose concentrations, which suggests that the regulation, production, and secretion of omentin might differ in animals than in humans. More data are needed to validate this hypothesis.

Human studies have demonstrated that plasma omentin concentrations are reduced in obese individuals, likely due to inflammation and apoptosis of adipose tissue [15,49,50]. Reduction of plasma omentin levels has been significantly correlated with increased waist circumference, bodyweight, dyslipidemia, high blood pressure, and glucose intolerance [16,50,51]. Conversely, bodyweight loss induced elevation of serum omentin levels [52]. In obese people, chemerin which is positively associated with insulin, and low-density lipoprotein cholesterol, may have a role in insulin resistance and oxidative stress [53]. Another study reported significantly higher circulating chemerin and lower omentin levels in patients with nascent metabolic syndrome as compared to controls, and that these effects persist after adjustment for body mass index, waist circumference, and age [54]. A systematic review of animal and clinical studies was recently conducted to assess the association between overall dietary intake and omentin expression and circulation. The results indicated that a long-term diet with reduced fat content and a balanced distribution of fatty acids, i.e., a higher monounsaturated fatty acids and lower saturated fatty acid intake, may increase insulin sensitivity, reduce inflammation, and, consequently, effectively increase the plasma concentration of omentin, but more research is needed to consolidate these assumptions [15].

Regarding the bodyweight variable, our cluster analysis showed that the group of horses with low BCS and CNS values correlated well with the lowest bodyweight and the group with high BCS and CNS values correlated well with the highest bodyweight (*p* < 0.001). In addition, statistically significant differences were detected amongst the four resulting groups in terms of NEFA, chemerin and omentin plasmatic levels. From this point of view, cluster analysis supported the results provided by both Spearman correlation and univariate linear regression and provided a more detailed image of the relationships between the studied variables.

As one of the study limitations, the evaluation of the parameters was performed on horses within a single breed. This breed was selected given its availability and representativeness for Northwestern Romania and also due to its predisposition to develop obesity and related pathologies. Another limitation of the study refers to the lack of variation in body condition score found in the horses examined. These aspects involve the studied horse breed, with the local Romanian draft horses being at relative higher risk to develop obesity given the traditional feeding practices. Furthermore, this breed is characterized by a robust constitution required for working animals.

To overcome such limitations, not only Spearman correlation and univariate linear regression, but also hierarchical clustering was used to analyze the correlation between total cholesterol, insulin, NEFA, adipokines and age, bodyweight, general and local adiposity scores (BCS and CNS, respectively). Moreover, our findings suggest the need for longitudinal studies to explore the studied parameters biomarkers value in other breeds, allowing generalization in broader populations of horses.

## 5. Conclusions

In conclusion, our results obtained using univariate linear regression analysis and hierarchical clustering offer new insights into equine metabolic syndrome, pointing out low significant and positive correlations between plasmatic levels of NEFA, chemerin, and omentin and the morphological traits. Moreover, to the best of our knowledge, this is the first report regarding the positive association between the omentin plasmatic level and CNS in Romanian draft horses described with equine metabolic syndrome.

These findings highlight NEFA, chemerin, and omentin as valuable biomarkers that could be further analyzed in other horse breeds for a better understanding of equine metabolic pathology.

## Figures and Tables

**Table 1:** ELISA kits’ characteristics according to the manufacturers’ manuals.

Metabolic Parameters	Sensitivity	Detection Range	Inter-Assay Coefficient of Variability	Intra-Assay Coefficient of Variability
Horse non-esterified fatty acid (NEFA)	1.0 µmol/L	10–600 µmol/L	<15%	<10%
Horse total cholesterol	0.026 mmol/L	0.05–30 mmol/L	<10%	<8%
Horse insulin	3.1 µIU/mL	6.25–400 µIU/mL	<12%	<8%
Horse omentin	1.0 ng/mL	3.12–100 ng/mL	<15%	<15%
Horse chemerin	5.0 ng/mL	31.2–1000 ng/mL	<15%	<15%

**Table 2:** Study group characteristics.

Parameter	Value
Number of horses (N)	71
Age (years)	9.0 (8.0; 12.5)
Sex, male (n, %)	27 (38.0%)
Bodyweight (kg)	600.0 (425.0; 700.0)
Body condition score	8.0 (7.0; 9.0)
Cresty neck score	4.0 (3.0; 5.0)
NEFA (μmol/L)	420.1 ± 82.8
Omentin (ng/mL)	14.3 (10.2; 16.6)
Chemerin (ng/mL)	283.0 (219.3; 348.7)
Total cholesterol (mmol/L)	2.9 (1.8; 3.3)
Insulin (μIU/mL)	55.4 (40.4; 77.8)

n, number; %, percentage. Data were expressed as mean ± standard deviation, median (25th, and 75th percentiles), or numbers and percentages.

**Table 3:** Correlation of NEFA, total cholesterol, insulin, omentin, and chemerin levels with selected factors.

	NEFA		Total Cholesterol		Insulin		Omentin		Chemerin	
	*r*	*p*-Value	*r*	*p*-Value	*r*	*p*-Value	*r*	*p*-Value	*r*	*p*-Value
Bodyweight	0.322	0.006	0.262	0.027	0.066	0.587	0.213	0.075	0.157	0.192
Age	−0.271	0.022	−0.01	0.995	−0.176	0.143	0.078	0.520	−0.177	0.147
Body condition score	0.295	0.013	0.187	0.117	0.063	0.602	0.197	0.099	0.204	0.087
Cresty neck score	0.267	0.024	0.172	0.152	−0.021	0.864	0.234	0.049	0.212	0.076

NEFA, non-esterified fatty acid; r, correlation coefficient.

**Table 4:** Univariate linear regression analysis.

Model	Variable	Unstandardized Coefficients		Standardized Coefficients	*p*-Value
		B	SE	Beta	
**Model 1**	(Constant)	380.09	52.40		0.000
	Bodyweight	0.139	0.056	0.287	0.016
	Sex	−9.487	16.049	−0.069	0.556
	Age	−2.126	1.863	−0.131	0.258
**Model 2**	(Constant)	354.26	58.74		0.000
	Cresty neck score	31.83	12.401	0.297	0.013
	Sex	−18.783	15.863	−0.136	0.241
	Age	−2.700	1.865	−0.167	0.152

SE, standard error.

**Table 5:** Comparison of means between groups by the one-factor ANOVA method.

Variable	Group	*N*	*m*	*σ*	*F*(3, 55)	*p*
Age	1	29	10.69	2.67	1.854	0.148
	2	15	10.07	5.86		
	3	5	6.10	3.75		
	4	10	12.30	7.99		
Bodyweight (kg)	1	29	495.69	152.54	7.310	<0.001
	2	15	641.67	130.22		
	3	5	586.40	154.03		
	4	10	720.20	137.21		
NEFA (μmol/L)	1	29	381.14	46.11	20.961	<0.001
	2	15	486.46	35.87		
	3	5	370.47	57.59		
	4	10	468.40	63.67		
Omentin (ng/mL)	1	29	12.04	3.65	9.492	<0.001
	2	15	12.90	3.90		
	3	5	13.34	4.85		
	4	10	19.24	3.08		
Chemerin (ng/mL)	1	29	250.02	54.96	23.952	<0.001
	2	15	232.93	97.05		
	3	5	500.40	40.73		
	4	10	399.37	110.82		
Total cholesterol (mmol/L)	1	29	3.02	0.80	3.427	0.023
	2	15	2.71	1.31		
	3	5	1.63	0.33		
	4	10	2.50	0.80		
Insulin (μIU/mL)	1	29	50.57	26.40	0.494	0.688
	2	15	55.89	29.51		
	3	5	62.63	38.43		
	4	10	60.03	20.29		

**Table 6:** Results of Kruskal–Wallis test used to compare BCS and CNS ranks.

Group	Ranks Average	
	BCS	CNS
Group 1	22.69	22.09
Group 2	35.03	36.90
Group 3	29.60	32.70
Group 4	43.85	41.25
χ^2^(3) *p*	14.715 0.002	14.671 0.002

**Table 7:** Spearman correlation rank coefficients between BCS, CNS, and bodyweight.

		BCS	CNS
Bodyweight	*ρ*	0.944	0.745
	*p*	<0.001	<0.001
BCS	*ρ*	1.000	0.704
	*p*		<0.001

## Data Availability

All data collected and analyzed during this study are included within the article.
